# Prevalence of common mental health issues among migrant workers: A systematic review and meta-analysis

**DOI:** 10.1371/journal.pone.0260221

**Published:** 2021-12-02

**Authors:** Siti Idayu Hasan, Anne Yee, Ariyani Rinaldi, Adlina Aisya Azham, Farizah Mohd Hairi, Amer Siddiq Amer Nordin

**Affiliations:** 1 Nicotine Addiction Research Group, Wisma R & D Universiti Malaya, University of Malaya Centre of Addiction Sciences, Kuala Lumpur, Malaysia; 2 Universiti Malaya Centre for Community & Sustainability, University of Malaya, Kuala Lumpur, Malaysia; 3 Department of Psychological Medicine, Faculty of Medicine, University Malaya, Kuala Lumpur, Malaysia; 4 Department of Social & Preventive Medicine, Faculty of Medicine, University Malaya, Kuala Lumpur, Malaysia; Universidad del Desarrollo, CHILE

## Abstract

Previous literature has shown that migrant workers manifested higher common mental issues (especially depressive symptom) compared to local workers due to stressors such as financial constraint and lack of access to healthcare. The aim of this systematic review and meta-analysis is to summarize the current body of evidence for the prevalence of depression and anxiety among migrant workers as well as exploring the risk factors and the availability of social support for migrant workers. Seven electronic databases, grey literature and Google Scholar were searched for studies from 2015 to 2021 related to mental health, social support and migrant workers. Study quality was assessed using the Newcastle Ottawa Scale and the Joanna Briggs Institute Qualitative Assessment and Review Instrument (JBI-QARI). Study heterogeneity was evaluated using *I*^2^ statistics. Random effects meta-analysis results were presented given heterogeneity among studies. The search returned 27 articles and only seven studies were included in meta-analysis, involving 44 365 migrant workers in 17 different countries. The overall prevalence of depression and anxiety among migrant workers was 38.99% (95% CI = 0.27, 0.51) and 27.31% (95% CI = 0.06, 0.58), respectively. Factors such as age, biological (health issue, family history of psychiatric disorder), individual (poor coping skills), occupational (workplace psychosocial stressors, poor working condition, salary and benefits issue, abuse), environmental (limited access towards healthcare, duration of residence, living condition) and social factor (limited social support) were associated with a mental health outcome in migrant workers. The availability of social support for migrant workers was mainly concentrated in emotional type of support. A high prevalence of depression and anxiety was found among migrant workers across the globe. This finding warrants a collective effort by different parties in providing assistance for migrant workers to promote their mental well-being.

## Introduction

Globalization has improved the interconnectedness between countries which has impacted human mobility in the aspect of migration [1, 2]. According to the International Organization for Migration (IOM) (2019), the number of international migrants has increased significantly from 84 million in 1970 to 272 million in 2019. In 2019, the highest two regions that hosted the total global international migrant stock were Europe and Asia, while Oceania, North America and Europe were the highest when a comparison made based on the size of the population in each region. Nearly half of the number of international migrants around the globe is originated from Asian countries (e.g., India, China, Bangladesh) followed by Mexico and the Russian Federation [3].

In 2017, the migrant workers’ population was still highly concentrated in higher-income countries (68%). However, due to economic development and issue related to immigration regulation in higher-income countries, there was an evident shift in the residence of migrant workers in middle-income countries. In terms of gender composition, the number of male migrant workers (58%) was higher than female migrant workers (42%) with apparent gender imbalance geographically in several regions such as the Arab States, where male migrant workers were highly demanded as the labor opportunities were more concentrated in the construction sector [3].

It is known that migrant workers commonly hired for jobs related to 3Ds (dangerous, dirty, difficult) [4, 5] or precarious employment which increased their exposure to environmental hazards at work site. They were at higher risk of workplace injuries due to inadequate safety protection at the workplace [6, 7]. Migrant workers were also reported to have poor working conditions such as low wages, higher working hours, low job security and workplace abuse [8]. Both occupational hazard and poor working environment have increased the vulnerability of migrant workers to poor health outcomes, especially on their mental well-being. Previous research indicated that migrant workers reported experiencing higher mental health problems in comparison to native workers [9–12].

The most prevalent mental health issues reported among migrant workers are the manifestation of depressive symptoms [13–17]. A systematic review assessing the prevailing psychological disorders among migrant workers also found that these workers were experiencing other issues such as anxiety, alcohol or substance abuse and poor sleep quality [17]. This psychological distress experienced by the migrant workers are commonly linked to several stressors: financial difficulties, health risks (due to working condition), limited access to healthcare and presence of social issue (i.e., language barrier, discrimination) [12, 13, 18–21]. A previous study has indicated the difference in stressors experienced by migrant workers according to gender and working industry [14]. Furthermore, World Health Organization (WHO) and International Labour Organization (ILO) has also highlighted the mental health impact of COVID-19 on migrant workers around the world, which mainly due to social isolation and job insecurity [22–24].

Looking for a potential solution to address the issue of mental health among migrant workers is a frame of reference in the discussion of protection on their welfare. One of the protective factors that may promote mental well-being of migrant workers is social support. Social support has been found to promote the mental well-being [25, 26] including in immigrants [27, 28] and refugees [29]. According to the traditional theoretical framework of social support, four types of social support were identified: emotional support (i.e., expressions of love, trust, and empathy), instrumental support (i.e., tangible aid and service), informational support (i.e., advice, suggestion) and esteem (i.e., useful information for self-evaluation) [30]. These difference in dimensions of social support may provide another focus for the intervention of the psychological well-being of the migrant workers.

Hence, in addressing the concern of mental well-being among migrant workers, this paper aims to conduct a systematic review of literature and meta-analysis in examining the prevalence of mental health issue (i.e., depression and anxiety) among migrant workers, as well as determining the risk factors of mental health outcomes and exploring the availability of social support for migrant workers.

## Method

This systematic review was conducted according to the Preferred Reporting Items for Systematic Reviews and Meta-Analyses (PRISMA) guidelines [31]. The protocol of this systematic review was registered with PROSPERO (protocol ID: CRD42021232181).

### Search strategy and selection criteria

A systematic search of all English-language literature published from 2015 to 2021 from MEDLINE, Education Research Complete, Psychology and Behavioral Sciences Collection, ERIC, SAGE, Science Direct, Scopus and Google Scholar search was performed. The following keywords (a) mental health or mental illness or mental disorder or psychiatric illness (b) anxiety (c) depression or depressive disorder or depressive symptoms or major depressive disorder (d) social support or social networks or social relationships or social inclusion or social exclusion or social isolation and, (e) migrant workers were used. The screening process in this review also included references of the selected articles, book chapters, papers presented at conferences, dissertations, editorial and commentaries. In addition, the authors of this paper attempted to contact the respective authors via email to obtain the full articles and detailed data if the articles were unavailable or information of the quantitative studies was inadequate.

Two independent reviewers performed all of the titles and abstracts screening, followed by an analysis of the full-text articles. All duplicates were removed. Any discrepancies were resolved by a third reviewer. Data from eligible studies were extracted by a reviewer and all extracted data were reviewed by two independent reviewers.

### Inclusion and exclusion criteria

Inclusion and exclusion criteria were set to identify and choose the studies that were most relevant to our research.

The inclusion criteria were the following:

- Any study design (quantitative, qualitative, mixed-methods studies)
- Published between 2015 and 2021
- Published in English
- Any migrant workers

The exclusion criteria were the following:

- Published prior to 2015
- Published in a language other than English
- Internal/external migrants moving within the same country

### Quality assessment

Study quality was assessed using the appropriate appraisal tool for each research design: the Newcastle Ottawa Scale (NOS) for Cohort studies [32], the Newcastle Ottawa Scale adapted for Cross-Sectional Studies [33] and the Joanna Briggs Institute Qualitative Assessment and Review Instrument (JBI-QARI) [34]. For mixed-method studies, the quality assessment was conducted based on the data used in this review. If both quantitative and qualitative data were included in this review, both the Newcastle Ottawa Scale and JBI-QARI were used to conduct the quality assessment, while if only one, the study quality was evaluated using either any one of the tools (based on the data used). It was performed by two reviewers and any discrepancies were discussed with the third reviewer.

### Statistical analysis

All data analyses were performed using Stats Direct (version 2.7.9). The presence of heterogeneity between the trials was tested using the I-squared (*I*^2^) statistic. An *I*^2^ of more than 75% indicated significant heterogeneity. If the *I*^2^ was significant, pooled prevalence of anxiety and depression were calculated by using a random-effects model [35]. Conversely, the data were pooled by using a fixed-effects model [36, 37]. Publication bias was assessed with the Begg-Mazumdar and Egger test. Qualitative meta-analysis was also conducted to summarize, compare and contrast the extracted data.

### Ethics approval

Ethical approval was obtained from Universiti Malaya Research Ethics Committee (UMREC) (UM.TNC2/UMREC_1187).

## Results

**Fig 1:**
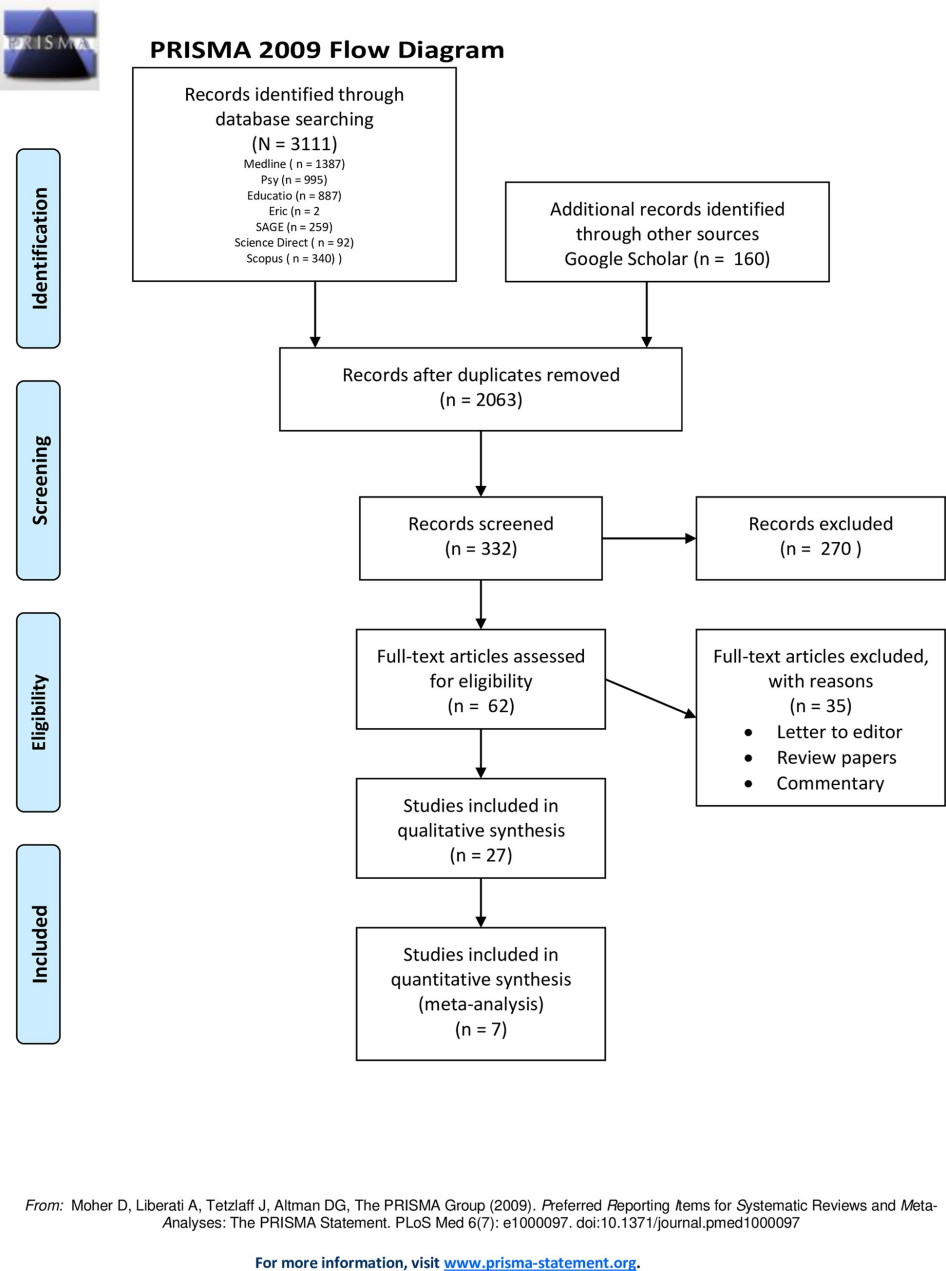
PRISMA flow chart.

Electronic database searching identified a total of 3962 articles. Additional literature was also identified using Google Scholar with 160 articles. After removal of duplicated publication, screening of title and abstract and screening of full-text, 27 studies were included in the present systematic review (see S1 Table). Out of these 27 studies, only seven articles had the data on the proportion of depression and anxiety for meta-analysis. Fig 1 shows the data extraction conducted in accordance with the Quality of Reporting of Meta-analyses Guidelines [31].

### Study characteristics

Most of the literature included were cross-sectional studies (18), followed by longitudinal studies (4), mixed-method studies (3) and qualitative studies (2). Participants were recruited using various sampling method including convenience sampling (7), purposive sampling (5), random sampling (5), snowball sampling (4), cluster sampling (2), stratified multi-stage probability proportional to size (1), cluster and occasional sampling (1), systematic random sampling and purposive (1) and convenience and snowball sampling (1).

Five of the studies were conducted in Australia [38–42]. Two studies were conducted in each of these countries: Singapore [43, 44], Hong Kong [45, 46], Italy [47, 48], The United States of America [49, 50], Spain [51, 52] and United Kingdom [53, 54]. The remaining studies were conducted in Israel [55], India [56], South Korea [57], Malaysia [58], Thailand [59], Japan [60], Norway [61], Ethiopia [62], Chile [63] and China [64].

There were 44 365 subjects included from the 27 studies (see S1 Table) with a sample size ranged between 40 and 15 321. Only seven studies reported the mean age of the participants, which range from 28.17 to 43.60 years [43, 44, 50, 53, 56, 59, 64] Out of 24studies that reported on gender composition, 13 studies had more female participants while another 11 studies had more male participants. Five studies did not state any information on the gender composition of their participants [43–46, 50].

In total, 39 psychometric instruments were used in 24 studies. Another remaining three studies used interview questions [44, 45] and combination of digital audio recorder and notes on emotion non-verbal cues [49].

### Study quality

Table 1 showed summary of study quality for cross-sectional by using the NOS scale. The articles’ scores range from seven to nine stars. Of the 18 studies, 17 could be regarded as good quality [38, 39, 41–43, 46, 48, 54, 55–57, 59–64] and only one was scored to be of poor quality [58]. The summary of study quality for longitudinal studies assessed using the NOS scale was presented in Table 2. The articles’ score ranges from six to eight stars. Of the four studies, one could be regarded as good quality [40] and three were scored to be of poor quality [51–53].

**Table 1:** Summary of quality assessment using the Newcastle-Ottawa Scale for cross-sectional studies.

Cross-sectional studies									
		Selection				Comparability	Outcome		
No	Author	Representativeness of the sample	Sample size	Non-respondents	Ascertainment of the exposure	Subjects in different outcome groups are comparable	Assessment of the outcome	Statistical test	Total
1	Adebayo et al. (2020)	*	*		**	**	*	*	8
3	Anjara et al. (2017)	*	*	*	**	**	*	*	9
4	Attal et al. (2020)	*	*	*	**	**	*	*	9
6	Yeung et al. (2020)	*	*	*	**	**	*	*	9
7	Capasso et al. (2018)	*	*		**	*	*	*	7
8	Chen et al. (2019)	*	*	*	**	**	*	*	9
11	Daly et al. (2018)	*	*		**	**	*	*	8
12	Daly et al. (2019)	*	*	*	**	**	*	*	9
13	Dhungana et al. (2019)	*	*	*	**	**	*	*	9
14	Gambaro et al. (2020)	*	*	*	**	**	*	*	9
17	Hong & Lee (2019)	*	*		**	**	*	*	8
18	Htay et al. (2020)	*	*	*	**		*	*	7
19	Kesornsri et al. (2019)	*	*		**	**	*	*	8
20	Liu et al. (2020)	*	*	*	**	**	*	*	9
21	Martynowska et al. (2020)	*	*	*	**	**	*	*	9
22	Miller et al. (2020)	*	*	*	**	**	*	*	9
23	Organista et al. (2019)	*	*	*	**	**	*	*	9
24	Straiton et al. (2019)	*	*		**	**	*	*	8
26	Tilahun et al. (2020)	*	*	*	**	**	*	*	9
27	Urzúa et al. (2019)	*	*	*	**	**	*	*	9

Good quality: 3 or 4 stars in the selection domain AND 1 or 2 stars in comparability domain AND 2 or 3 stars in outcome/exposure domain.

Fair quality: 2 stars in selection domain AND 1 or 2 stars in comparability domain AND 2 or 3 stars in outcome/exposure domain.

Poor quality: 0 or 1 star in selection domain OR 0 stars in comparability domain OR 0 or 1 stars in outcome/exposure domain.

**Table 2:** Summary of quality assessment using the Newcastle-Ottawa Scale for longitudinal studies.

Longitudinal studies										
		Selection				Comparability	Outcome			
No	Author	Representativeness of exposed cohort	Selection of the non- exposed cohort	Ascertainment of exposure	Demonstration that outcome of interest was not	Comparability of cohorts on the basis of the design or analysis	Assessment of outcome	Was follow- up long enough for	Adequacy of follow up of cohort	Total
9	Chen et al. (2019)	*	*	*	*	**		*	*	8
15	González-Castro et al. (2020)	*	*	*	*	**		*		7
16	Hatch et al. (2016)	*	*	*		**		*		6
24	Ronda-Pérez et al. (2019)	*	*	*		**		*		6

Good quality: 3 or 4 stars in the selection domain AND 1 or 2 stars in comparability domain AND 2 or 3 stars in outcome/exposure domain.

Fair quality: 2 stars in selection domain AND 1 or 2 stars in comparability domain AND 2 or 3 stars in outcome/exposure domain.

Poor quality: 0 or 1 star in selection domain OR 0 stars in comparability domain OR 0 or 1 stars in outcome/exposure domain.

For qualitative studies, most of the studies met the criteria of the JBI-QARI except for (1) the indication of locating the researcher culturally or theoretically (Criteria 6) and (2) the indication of the influence of the researcher on the research (and vice-versa) (Criteria 7) (see Table 3). For Criteria 7, only two studies met the criteria [44, 49].

**Table 3:** Summary of quality assessment using the JBI-QARI.

Checklist questions	Study 5 (Baig & Chang, 2020)	Study 10 (Crocker, 2015)	Study 26 (Tilahun et al., 2020)	Study 28 (Van Bortel et al., 2019)
1. Is there congruity between the stated philosophical perspective and the research methodology?	Yes	Yes	Yes	Yes
2. Is there congruity between the research methodology and the research question or objectives?	Yes	Yes	Yes	Yes
3. Is there congruity between the research methodology and the methods used to collect data?	Yes	Yes	Yes	Yes
4. Is there congruity between the research methodology and the representation and analysis of data?	Yes	Yes	Yes	Yes
5. Is there congruity between the research methodology and the interpretation of results?	Yes	Yes	Yes	Yes
6. Is there a statement locating the researcher culturally or theoretically?	No	No	No	No
7. Is the influence of the researcher on the research, and vice- versa, addressed?	No	Yes	No	Yes
8. Are participants, and their voices, adequately represented?	Yes	Yes	Yes	Yes
9. Is the research ethical according to current criteria or, for recent studies, and is there evidence of ethical approval by an appropriate body?	Yes	Yes	Yes	Yes
10. Do the conclusions drawn in the research report flow from the analysis, or interpretation, of the data?	Yes	Yes	Yes	Yes

### Meta-analysis

**Fig 2:**
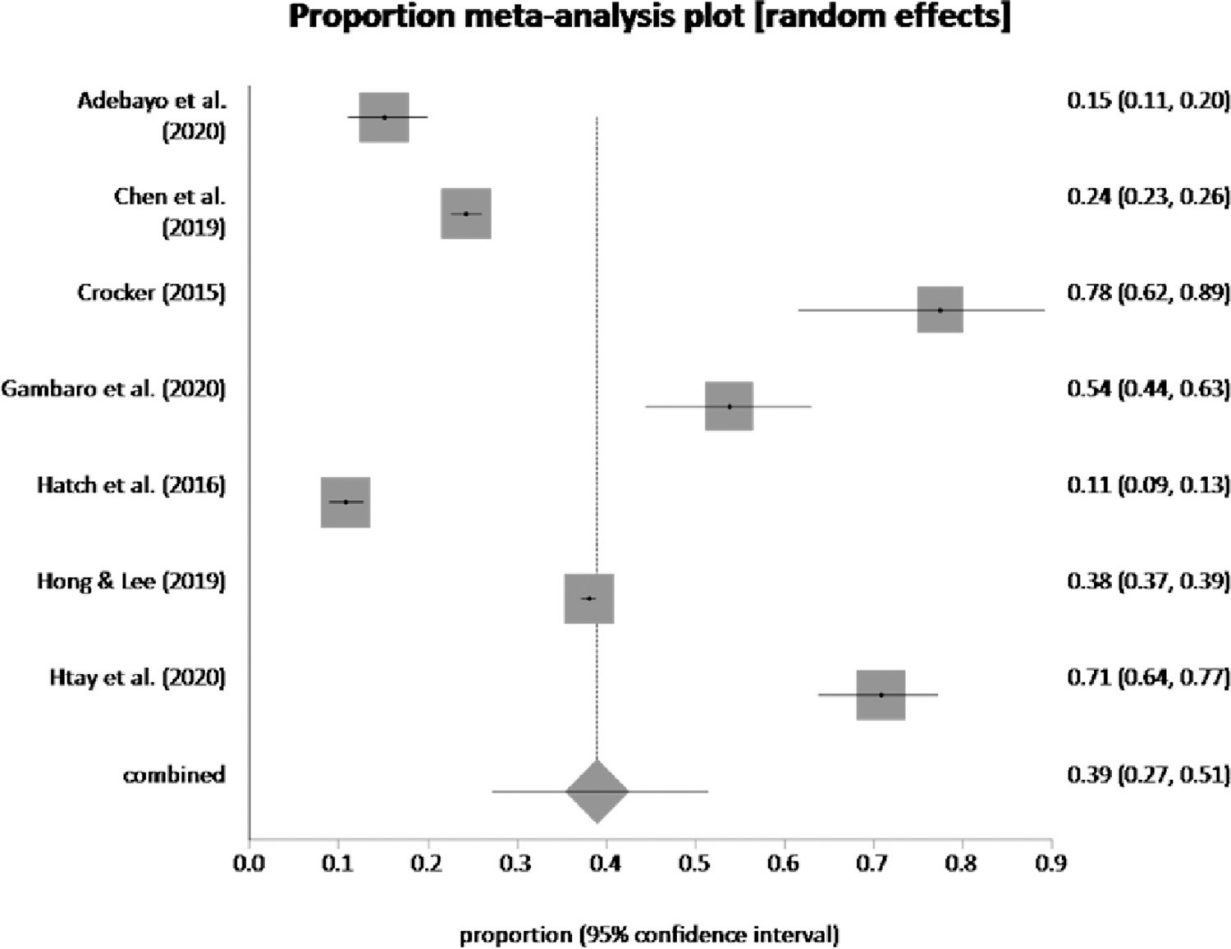
Pooled prevalence of depression by random effects meta-analysis is 38.99%. (95% CI = 0.27 to 0.51), I^2^ (inconsistency) = 99.2% (95% CI = 99.1% to 99.3%), Egger: bias = -1.679764 (95% CI = -21.867931 to 18.508402), P = 0.84, Begg-Mazumdar: Kendall’s tau.

**Fig 3:**
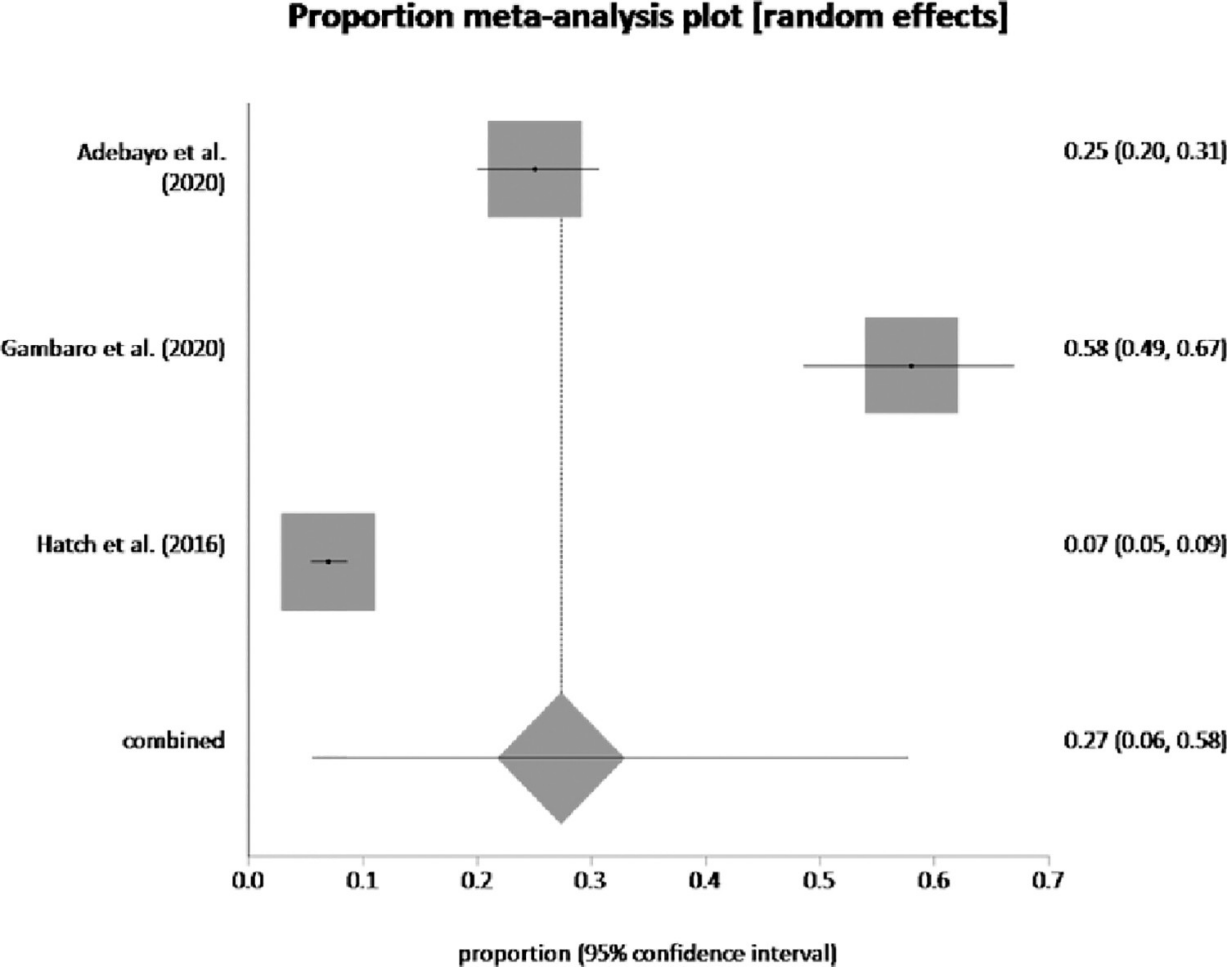
Pooled prevalence of anxiety by random effects meta-analysis is 27.31%. (95% CI = 0.06 to 0.58), I^2^ (inconsistency) = 98.9% (95% CI = 98.5% to 99.2%), Egger bias = <too few strata> (95% CI = * to *), P = *, Begg-Mazumdar: Kendall’s tau = <too few strata.

Seven studies assessed the prevalence of depression and anxiety which ranged from 10.7% to 85% for the former and 6.9% to 58.47% for the latter. Pooled proportion of depression was 38.99% (95% CI = 0.27, 0.51) (see Fig 2) and pooled proportion of anxiety was 27.31% (95% CI = 0.06, 0.58) (see Fig 3).

### Qualitative meta-analysis

#### Depression

Nine studies explored the presence of depression among migrant workers [41, 48–50, 57, 58, 64, 65]. The prevalence of depression among Myanmar migrant workers in Malaysia was 70.8% (N = 136) [58]. Chen et al. [64] found the prevalence of depression among migrant workers in China was 24.3%. In the study conducted by Hatch et al. [53], 10.7% of the respondents reported experiencing depressive episodes. Organista et al. [50] evaluated the challenging working and living conditions and the psychological distress in Latino migrant workers. Their study found a mean of 0.77 (SD = 0.54) for depressive symptoms, based on standardized factor loadings of 1.00. In another study [64], the mean of depressive symptoms reported by participants was 32 (SD = 9.6, range = 10–50).

In the semi-structured interview conducted by Crocker [49], the reported depressive symptoms were 70% among male participants (N = 14) and 85% among female participants (N = 17). While in the study conducted by Hong & Lee [57], the prevalence of depression was higher in migrants from low-income countries compared to migrants from middle- or high-income countries (male = 27.1% > 23%; female = 37.1% > 31.2%).

Adebayo et al. [41] investigated the prevalence of acculturation stress and mental health issue (i.e., depression, anxiety, stress) among migrant workers in Australian residential aged care facilities. Based on Depression Anxiety Stress Scale-21 (DASS-21), their results found that 84.7% of the migrant workers in the normal range. Only 1.8% and 0.8% were in the range of severe depression and extremely severe depression, respectively.

Similarly, Gambaro et al. [48] found that 42.37% of the migrant workers in Italy reported no depressive symptoms, based on the Zung Self-Rating Depression Scale (SDS). 38.14% of the participants were in the low range, 14.41% in the mild range and only 1.69% in the high range.

#### Anxiety

Four studies assessing the presence of anxiety among migrant workers were identified [41, 48, 50, 53]. Hatch et al. [53] reported 6.9% of their participants manifested generalized anxiety disorder (GAD). In another study, Organista et al. [50] reported a mean of 0.66 for anxiety symptoms (SD = 0.60), based on standardized factor loading of 1.00.

Adebayo et al. [41] found in their studies that 74.5% of the migrant workers were in the normal anxiety range of DASS-21. Only 2.3% and 3.8% were in the range of severe anxiety and extremely severe anxiety, respectively. In another study [48], 38.98% of the participants reported no symptoms of anxiety, based on the rating on Zung Self-Rating Anxiety Scale (SAS). 49.15% of the migrant workers were in the low range and another 9.32% were in the mild range of anxiety.

#### Stress

Five studies explored the prevalence of stress (including acculturation stress and post-traumatic stress disorder) among migrant workers [41, 43, 48, 49, 54].

The prevalence of stress among female migrant domestic workers in Singapore was 52.5% (N = 85) [43]. Crocker [49] found in his study that 95% of male participants (N = 19) and 80% of female participants (N = 16) reported feeling stressed. In another study [54], the mean of perceived stress among the respondents was 16.89 (SD = 7.19). Adebayo et al. [41] revealed in their study that based on the DASS-21, 89.9% of the migrant workers were in the normal range. Only 1.8% of the workers reported being on the severe level of stress scale. The study also evaluated the prevalence of acculturation stress among migrant workers in Australia. It was found that the mean of acculturation stress was 38.4 (SD = 14.1).

In the study conducted by Gambaro et al. [48], based on PTSD Checklist for DSM-5 (PCL-5), 53.95% (N = 63) reported scoring above the median score of 33 which suggested higher level of post-traumatic stress disorder symptoms.

### Other mental health outcome

Three studies identified the prevalence of common mental health issues (both depressive and anxiety symptoms) [47, 59, 61]. Two studies measured the mental well-being of migrant workers using the Hopkins Symptoms Checklist-25 (HSCL-25) [59, 61]. The prevalence of participants with depressive and anxiety symptoms was 12.7% [61] and 11.9% [59]. In another study by Capasso et al. [47], the prevalence of the anxious-depressive disorder among migrant workers was 32.8%.

### Risk factors for mental health outcome

#### Age

Six studies found the effect of age on the mental well-being of the migrant workers [43, 55–57, 61, 64]. Chen et al. [64] reported in their findings that the prevalence of mental health issue was higher among younger group of migrant workers (25–39 years old) (M = 6.96, SD = 4.67, p = .008). Similarly, age was found to inversely associated with the symptoms of anxiety and depression (OR = 0.95, p = .03) [55]. Another study also found that older age (> 50 years old) was correlated with less psychological distress (M = 78.2, SD = 11.9, p < .001) [43].

In contrast, a study found that older age group (> 45 years old) was associated with higher psychological distress (aPR = 2.74, CI = 1.01–7.41, p < .05) [56]. Straiton et al. [61] also reported in their study that the older age group experienced more mental health issue (16.0% vs 11%, p < .001). In addition, the prevalence of depressive symptoms was found to be higher in immigrants aged above 65 years old (40.5%) [57].

#### Psychological factors

Several studies explored the association between the coping skills of migrant workers and their mental well-being [48, 52]. Resilience among migrant workers was found to inversely associated with depressive symptoms (r = -0.24, p < .05) and suicidal intention (r = - 0.31, p < .05) [48]. In addition, lower attention to feelings (i.e., thinking about one’s feelings) (standardized path coefficient = 0.38 (T1), 0.49 (T2), p ≤ .01) and higher mood repair (i.e., one’s ability to regulate moods when experiencing negative emotions) (standardized path coefficient = -0.39 (T1), -0.41 (T2), p ≤ .01) were linked to better psychological well-being among migrants in Spain during both data waves [52].

In addition, migrant workers with higher negative affectivity (i.e., temperamental style characterized by stable tendency to experience negative emotions) (OR = 1.961, p < .05) and higher social inhibition (OR = .343, p < .05) were associated with higher level of anxious-depressive disorder [47].

#### Biological factors

Two studies found the biological risk factors that influenced the mental health outcome among migrant workers [56, 62]. Higher psychological distress was reported among migrant workers with existing health issues (aPR = 2.0, p < .001) [56] and history of psychiatric illness in their family (OR = 6.75, 95% CI = 1.03, 43.95) [62].

#### Occupational factors

Daly et al. [39] reported that higher psychological distress among migrant workers in Australia was associated with workplace psychosocial stressors including complex/demanding jobs (OR = 2.6, p < .0001), jobs with low control (OR = 1.8, p < .0001), jobs with low security (OR = 3.4, p < .0001) and overall job adversity (OR = 2.7, p < .0001). In a similar study, they found that higher mental well-being was associated with higher skill discretion (OR = 0.40, p < .001), higher decision authority (OR = 0.36, p < .001) and lower job insecurity (OR = -1.08, p < .001) [42].

A study found that the effect of occupational roles on acculturation stress among migrant workers in Australia [41]. It was described that in comparison to other roles in registered aged care facilities, enrolled and registered nurses reported higher level of acculturation stress (F(3, 254) = 3.0, p = .03).

In the study conducted by Organista et al. [50], poor working conditions (measured by working days, working hours and earnings) were associated with higher level of desesperación (i.e., feeling of isolation) (β = -0.10, p ≤ .01) and depression (β = -0.11, p ≤ .01). Miller et al. [60] found in their study that higher employment satisfaction was associated with better mental health outcome among migrants working in Japan (B = 4.9; p < 0.001). In addition to poor working conditions, experiencing physical abuse at workplace was also found as a risk factor of psychological distress among migrant workers (OR = 12.17, 95% CI = 5.87, 25.22) [62].Several studies also reported the risk of mental health issues among migrant workers when experiencing an occupational issue related to salary and benefits [56, 58, 62]. Myanmar migrant workers in Malaysia reported higher depressive symptoms when there was absence of financial aid from employer if they had physical health issue (80.9%, p = .001) [58]. Similarly, receiving no sick leave (aPR = 2.4, p < .001) [56] and unable to get salary timely (OR = 3.35, 95% CI = 1.47, 7.63) [62] were also associated with the presence of psychological distress among migrant workers.

#### Environmental factors

Two studies identified having limitation towards healthcare access in the working country as one of the risk factors of mental health issues among migrant workers [56, 62]. Higher presence of common mental disorder symptoms was reported by Ethiopian labour migrant returnees working in Middle East countries when they were denied access to healthcare (OR = 3.20, 95% CI = 1.53, 6.67) [62]. In another study [56], the risk of psychological distress was identified among Nepali migrant workers in India who experienced barriers to healthcare access (aPR = 1.88, p < .001).

Duration of residence was also found to associate with the mental well-being among migrant workers. Htay et al. [58] indicated in their study that migrant workers who had lived in Malaysia for a duration of five years and more were found to manifest more depressive symptoms (81.9%, p < .001) in comparison to those who stayed less than five years. Similarly, migrant workers who had stayed in Spain for more than 10 years also reported higher incidence of common mental health problem compared to those with shorter residence duration in Spain (1–10 years) (ORa = 0.06, 95% CI = 0.26–0.01) [51].

Furthermore, poor living condition in migrant workers was linked to the depression (β = −0.17, SE = 0.02, p ≤ .001), desesperación (i.e., feeling of isolation) (β = −0.19, SE = 0.03, p ≤ .001) and alcohol use (β = −0.13, SE = 0.43, p ≤ .01) [50].

#### Risk factors of mental health outcome during COVID-19

Two studies explored the risk factors of the mental health outcome among migrant workers during COVID-19 [46, 55]. In the study by Attal et al. [55], they found that emotional distress among the workers was associated with household food insecurity (OR = 5.85, p < .001), lower in confidence to care for themselves and employer during COVID-19 (OR = 3.85, p < .001), poorer general health (OR = 2.98, p < .003), country of origin (i.e., those who were not from Philippines) (OR = 2.83, p < .001) and gender (i.e., female) (OR = 2.34, p < .04). In another study, the anxiety symptoms experienced by Filipino domestic helpers in Hong Kong was associated with lack of protective equipment (OR = 1.54, p = .00), higher workload during pandemic (OR = 1.95, p = .00) and worried about termination if getting COVID-19 (OR = 1.43, p = .00) [46].

#### Social factors/social support

The feeling of isolation was associated with higher psychological distress outcome [43, 60]. Female migrant domestic workers in Singapore with higher level of stress was associated with feeling isolated (M = 17.6, SD = 3.4) [43]. Migrants working in Japan reported that lower feeling of isolation promoted their mental well-being (B = 3.2, p = .008) [60].

In the study conducted by Straiton et al. [61], mental health issues were found higher among those without social support (20%) in comparison to those with social support (11.2%). A qualitative study conducted by Van Bortel et al. [44] reported that the respondents indicated social support was an important coping resource for them with two themes identified which were the comfort of company and having someone to talk to. In contrast, in the study conducted during COVID-19, it was revealed that no association was found between social support from employers, family, friends and community organizations and anxiety symptoms (OR = 1.21, p = 0.16) [46].

In the study conducted by Baig & Chang [45], their qualitative data described the difference in help-seeking behaviour among migrant domestic workers in Hong Kong between formal and informal support systems. It was found that the workers approached the formal social support (e.g., consulate, local government departments, agency) for the issue related to employment. On the other hand, these migrant workers preferred to seek informal social support (e.g., family, friends) when experiencing emotional distress. Another qualitative data found that limited formal social support (from established organization) was provided for Ethiopian migrant workers returnees from Middle East countries who experienced mental health issues [62]. Only three organizations were reported to provide formal social support (in terms of mental health care services: two of them provided a rehabilitation center and another one provided hospital-based mental health care.

## Discussion

The present systematic review and meta-analysis included a total of 27 studies, with most studies being cross-sectional. The review of studies has fulfilled the aim of identifying the mental health issue among migrant workers and their risk factors. Our meta-analysis literature review highlights the pool prevalence of depression and anxiety among migrant workers. The prevalence of depression and anxiety was 38.98% and 27.31%, respectively. There was a notable increase in the prevalence in comparison to a decade ago where the reported prevalence of depression and anxiety among labour migrants was 20% and 21%, respectively [66]. This could be influenced by the increasing trends of working-related migration in the last ten years [3].

It was also found that refugees had a similar prevalence of depression and anxiety, with 40.9% and 26.6%, respectively [67]. Another study reported a wide variation in prevalence rates for depression and anxiety among first-generation migrants with, 5% to 44% and 4% to 40%, respectively [68]. This shows that migration in general amplified the risk for an individual to experience mental health issues. However, the wide variation noted in the study between first-generation migrants may highlight the difference of the postmigration environment between migrant workers, refugees and first-generation migrants which warrants further investigation.

This review also identified the prevalence of stress, including acculturation stress and post-traumatic stress disorder, among migrant/migrant workers. Most of the studies found a high prevalence of stress, except for Adebayo et al. [41]. In that study, migrant healthcare workers working in aged care residential facilities reported a higher level of acculturation stress in comparison to general stress. Acculturation stress is known to be a common issue among migrant labours [69].

### Risk factors associated with mental health issues among migrant workers

Our review had identified numerous factors associated with mental health issues among migrant workers including age, psychological factors, occupational factors, biological, environmental factors and social factors. Firstly, there were no age group differences in terms of its influence on mental well-being. This could be due to the methodological heterogeneity across studies, which restricted this review to make an inference regarding the age difference.

Psychological factors were found to be associated with mental health issues among migrant workers. Migrant workers with higher resilience and a higher level of emotional intelligence (i.e., good perception, understanding and management of their feelings) reported a higher level of psychological well-being. Resilience and emotional intelligence are known as protective factors that can contribute to better psychological health as it helps an individual to adapt and to regulate their emotions better during stressful situations [70–72]. Personality traits (i.e., negative affectivity and social inhibition) were also linked with the mental well-being of migrant workers, where those who did not display the traits reported a higher level of mental well-being. This is consistent with previous literature [73–75]. These findings on the relationship between migrant workers’ mental well-being and their resilience, emotional intelligence and personality traits suggest that intervention may focus on a psychological aspect such as building resilience and emotional regulation to promote mental well-being among migrant workers.

Biological factors, particularly family psychiatric history and existing health conditions, have also been identified as risk factor for mental health issues among migrant workers. healthcare service. It is established that the likelihood for an individual to experience mental health issue is higher when they have a family member/relative who is diagnosed with a psychiatric disorder in comparison to those with no family history [76]. This also may be moderated and/or mediated by other factors including stressful life events, poor coping skills and lack of social support [77]. Moreover, it is interesting to highlight the presence of existing health conditions in migrant workers together with another risk factor found which was the barriers to healthcare access. Migrant workers are common experiencing financial limitations and a lack of formal medical protection which restricted them from seeking formal healthcare service [78–80]. This barrier will put a constraint on health-seeking behaviour among migrant workers, including those with existing health issues.

In addition to barriers to healthcare access, other environmental factors which were duration of stay and poor living conditions were noted as the risk factors of mental health issues among migrant workers. Our findings found that a longer duration of stay in the working country was related to the poorer mental health of migrant workers. Previous research has shown mixed findings of the relationship between migrant workers’ mental well-being and their length of stay at working country [81, 82] which suggests that other plausible factors (e.g., occupational, social, psychological) should be considered to explain this association. Concerning the poor living condition as another risk factor, this aspect has been highlighted by Organisation for Economic Co-operation and Development (OECD) reports. A decent living environment is a socioeconomic indicator that influences the social integration of migrants in the host or working country and as a result, improves their general well-being [83, 84].

Our review identified the association between occupational stressors and mental health issues among migrant workers. The stressors include job characteristics, type of occupational role, poor working conditions, salary and benefits issues and physical abuse at the workplace. Migrant workers reported a poorer level of mental well-being due to job characteristics such as demanding jobs, jobs with low control and high job insecurity. Our findings were consistent with previous literature that discussed job characteristics as a predictor of mental health issues among employees in the general population [85–88]. Job characteristics have also been found to predict job satisfaction [89–91]. One of the studies in our review showed in their findings that employment satisfaction was related to higher mental well-being among migrants working in Japan [60]. This suggests that migrant workers’ job satisfaction and mental health are likely to be explained by the characteristics of their jobs, emphasizing the importance of evaluating each of these factors in maintaining the mental well-being of migrant employees.

On another note, Adebayo et al. [41] discussed the difference between occupational roles and acculturation stress (i.e., mental challenges of adapting to a new culture) where migrant nurses were found to report a higher level of acculturation stress. Migrant workers were known to be at higher risk to experience discrimination and communication problems at the workplace [20, 92, 93]. These issues may intensify the stress level of these nurses as they are working directly with the community which requires quick adaptation to the culture of their working country. As for the remaining occupational risk factors (poor working conditions, salary and benefits issues and physical abuse at the workplace), they can be seen as a result of a lack of labor rights protection for the migrant workers. All of the stated factors are identified by ILO as the common violations experienced by migrant workers [94, 95].

Several studies included in this review also identified the associated factors of mental health issues among migrant workers during COVID-19, which mainly related to job stressors such as lack of confidence to properly care for themselves and their employer, lack of protective equipment, higher workload and worries being terminated if contracting COVID-19. It is known that migrant workers had lower job security compared to local workers [96]. The job insecurity issue is likely to amplify during COVID-19 as the whole world is currently struggling economically. This is consistent with a qualitative study conducted among migrant workers from Bangladesh working in Southeast Asia and the Middle East regions [97]. The fear of losing jobs and worry about not getting a salary had taken a toll on their mental health, in which in several extreme cases, some of the migrant workers committed suicide.

Finally, social support was also linked with the presence of mental health issues among migrant workers. All of the four studies identifying the association measured the dimension of social support related to connectedness with others. Social disconnectedness has been established as a risk factor for psychological distress [98–101] which highlights the importance of assisting migrant workers to maintain socially connected with their family or friends in seeking emotional support to act as a coping resource for them. However, in contrast, a study conducted during COVID-19 indicated that social support was not associated with the symptoms of anxiety among migrant care workers [46]. This is inconsistent with previous studies that discussed the effect of lower social support and the presence of mental health issues among healthcare workers during COVID-19 [102, 103]. Different types of job characteristics between care workers and healthcare workers may have played a role in observing the difference.

Only two studies discussed the availability of material and informational support for migrant workers. Although emotional support is important in assisting migrant workers in promoting their mental well-being, having additional access to material and informational support may help the migrant workers to cope better with the exposed stressors. This may also increase the help-seeking behaviour in migrants with or at risk of psychiatric disorder.

### Strengths and limitations

This review has several strengths. Firstly, a comprehensive searching strategy was carried out in the literature sources, the grey literature and the reference lists of the eligible articles which allowed this review to capture a large number of studies. This review was also systematically conducted using the preferred reporting items criteria of PRISMA guidelines. The majority of the studies included in this review was identified to be of high quality. Furthermore, this study increased the theoretical knowledge on the associated risk factors of mental health issues among migrant workers. This information may act as a reference for the policymakers, authorities and employers to create preventive strategies for migrant workers.

However, there are limitations warrant consideration. Firstly, most of the studies included were conducted in cross-sectional, thus, no causal relationship can be established. Next, there is the presence of heterogeneity of the method and tools across the studies. There is a consideration that should be taken in interpreting the findings in this review. This review also only included study in the English language. Hence, some literature that meets the inclusion criteria may not have been reviewed.

## Conclusion

In summary, this review and meta-analysis have provided an overview of the mental health outcome among migrant workers. There is evidence of an increase in depressive and anxiety symptoms in this specific population and various risk factors were identified to associate with the mental health issue, including social support. It is recommended for future research to conduct more cohort and longitudinal studies in looking at the trend or progress of the mental health outcome associated with different factors including demographics, biological, psychological, environmental, occupational and social.

The high prevalence of mental health problems among migrant workers warrants the implementation of necessary intervention strategies in addressing this issue. This may be adopted from the guideline provided by WHO [104] in promoting mental health in refugees and migrant. The area of interventions mentioned in the guideline including endorsing social integration and reducing the gap of barriers towards healthcare. A specific social support program related to the maintenance of connection with their family members and friends in their origin country should also be designed to act as a coping resource for the migrant workers.

The employer can also play a role in addressing the mental health concern among migrant workers. Some of the strategies that can be taken by employer are acknowledgement of mental health issue as a workplace concern, development of preventive strategies and mental health policies at workplace and facilitate the workers with mental health problems in accommodating at workplace [105].

Finally, the role of policymakers is vital in tackling this issue of mental distress among migrant workers. Based on the scientific evidence available, policies on healthcare, including mental health, should be prioritized with detailed planning and evaluation to ensure the sustainability of the policy. Close collaboration with non-governmental organization (NGO) can also take place to ensure a more comprehensive discussion can be taken which may lead to the rapid implementation of policies.

## Supporting information

S1 Checklist (PDF)

S1 Table Study characteristics. (DOCX)

## References

[1] Czaika M, de Haas H. The globalization of migration: has the world become more migratory? Int Migr Rev. 2014 Jun;48(2):283–323. doi: 10.1111/imre.12095

[2] Colic-Peisker V. Globalization and migration. In Farazmand A, editor. Global encyclopedia of public administration, public policy, and governance. Switzerland: Springer International Publishing; 2017. pp. 1–7.

[3] International Organization for Migration. World Migration Report 2020. [Internet]. 2019 [cited 2021 May 8]. Available from: https://publications.iom.int/system/files/pdf/wmr_2020.pdf

[4] Orrenius PM, Zavodny M. Do immigrants work in riskier jobs? Demography. 2009 Aug;46(3):535–51. doi: 10.1353/dem.0.0064 ; PMCID: PMC2831347. 19771943 PMC2831347

[5] Premji S. "It’s Totally Destroyed Our Life": Exploring the pathways and mechanisms between precarious employment and health and well-being among immigrant men and women in Toronto. Int J Health Serv. 2018 Jan;48(1):106–127. doi: 10.1177/0020731417730011 Epub 2017 Sep 14. . 28906167

[6] Yanar B, Kosny A, Smith PM. Occupational health and safety vulnerability of recent immigrants and refugees. Int J Environ Res Public Health. 2018 Sep 14;15(9):2004. doi: 10.3390/ijerph15092004 ; PMCID: PMC6165099. 30223449 PMC6165099

[7] Hargreaves S, Rustage K, Nellums LB, McAlpine A, Pocock N, Devakumar D, et al. Occupational health outcomes among international migrant workers: a systematic review and meta-analysis. The Lancet Global Health. 2019 Jul;7(7):e872–e882. doi: 10.1016/S2214-109X(19)30204-9 31122905 PMC6565984

[8] Moyce SC, Schenker M. Migrant workers and their occupational health and safety. Annu Rev Public Health. 2018 Apr 1;39:351–365. doi: 10.1146/annurev-publhealth-040617-013714 Epub 2018 Jan 24. . 29400993

[9] Aalto AM, Heponiemi T, Keskimäki I, Kuusio H, Hietapakka L, Lämsä R, et al. Employment, psychosocial work environment and well-being among migrant and native physicians in Finnish health care. Eur J Public Health. 2014 Jun;24(3):445–51. doi: 10.1093/eurpub/cku021 Epub 2014 Mar 19. . 24648502

[10] Cayuela A, Malmusi D, López-Jacob MJ, Gotsens M, Ronda E. The impact of education and socioeconomic and occupational conditions on self-perceived and mental health inequalities among immigrants and native workers in Spain. J Immigr Minor Health. 2015 Dec;17(6):1906–10. doi: 10.1007/s10903-015-0219-8 . 25972123

[11] Pham KTH, Nguyen LH, Vuong QH, Ho MT, Vuong TT, Nguyen HT, et al. Health inequality between migrant and non-migrant workers in an industrial zone of Vietnam. Int J Environ Res Public Health. 2019 Apr 28;16(9):1502. doi: 10.3390/ijerph16091502 ; PMCID: PMC6539052. 31035337 PMC6539052

[12] Devkota HR, Bhandari B, Adhikary P. Perceived mental health, wellbeing and associated factors among Nepali male migrant and non-migrant workers: A qualitative study. J Migr Heal [Internet]. 2021;3(November 2020):100013. doi: 10.1016/j.jmh.2020.100013 34405181 PMC8352157

[13] Lam KK, Johnston JM. Depression and health-seeking behaviour among migrant workers in Shenzhen. Int J Soc Psychiatry. 2015 Jun;61(4):350–7. doi: 10.1177/0020764014544767 Epub 2014 Aug 3. . 25091494

[14] Meyer SR, Decker MR, Tol WA, Abshir N, Mar AA, Robinson WC. Workplace and security stressors and mental health among migrant workers on the Thailand-Myanmar border. Soc Psychiatry Psychiatr Epidemiol. 2016 May;51(5):713–23. doi: 10.1007/s00127-015-1162-7 Epub 2015 Dec 12. . 26661796

[15] Nadim W, AlOtaibi A, Al-Mohaimeed A, Ewid M, Sarhandi M, Saquib J, et al. Depression among migrant workers in Al-Qassim, Saudi Arabia. J Affect Disord. 2016 Dec;206:103–108. doi: 10.1016/j.jad.2016.07.037 Epub 2016 Jul 19. . 27472411

[16] Khaled SM, Gray R. Depression in migrant workers and nationals of Qatar: An exploratory cross-cultural study. Int J Soc Psychiatry. 2019 Aug;65(5):354–367. doi: 10.1177/0020764019850589 Epub 2019 May 26. ; PMCID: PMC6651615. 31130042 PMC6651615

[17] Mucci N, Traversini V, Giorgi G, Tommasi E, De Sio S, Arcangeli G. Migrant workers and psychological health: A systematic review. Sustain. 2020;12(1):1–28. doi: 10.3390/SU12010120

[18] Lecerof SS, Stafström M, Westerling R, Östergren PO. Does social capital protect mental health among migrants in Sweden? Health Promot Int. 2016 Sep;31(3):644–52. doi: 10.1093/heapro/dav048 Epub 2015 Jun 4. . 26048867

[19] Ang JW, Chia C, Koh CJ, Chua BWB, Narayanaswamy S, Wijaya L, et al. Healthcare-seeking behaviour, barriers and mental health of non-domestic migrant workers in Singapore. BMJ Glob Heal. 2017;2(2). doi: 10.1136/bmjgh-2016-000213 28589024 PMC5435267

[20] Schilgen B, Nienhaus A, Handtke O, Schulz H, Mösko M. Health situation of migrant and minority nurses: A systematic review. PLoS One. 2017 Jun 26;12(6):e0179183. doi: 10.1371/journal.pone.0179183 ; PMCID: PMC5484487. 28650981 PMC5484487

[21] Adhikary P, Sheppard ZA, Keen S, Teijlingen E van. Health and well-being of Nepalese migrant workers abroad. Int J Migr Health Soc Care [Internet]. Emerald; 2018 Jan 10;14(1):96–105. doi: 10.1108/ijmhsc-12-2015-0052

[22] International Labour Organization. Impact of COVID-19 on migrant workers in Lebanon and what employers can do about it. [Internet]. 2020 [cited 2021 October 12]. Available from: https://www.ilo.org/wcmsp5/groups/public/—arabstates/—robeirut/documents/publication/wcms_741604.pdf

[23] International Labour Organization. Experiences of ASEAN migrant workers during COVID-19. [Internet]. 2020 [cited 2021 October 12]. Available from: https://www.ilo.org/wcmsp5/groups/public/—asia/—ro-bangkok/documents/briefingnote/wcms_746881.pdf

[24] World Health Organization Europe. Promoting the health of migrant workers in the WHO European region during COVID-19. [Internet]. 2020 [cited 2021 October 12]. Available from: https://apps.who.int/iris/bitstream/handle/10665/336549/WHO-EURO-2020-1384-41134-55925-eng.pdf?sequence=1&isAllowed=y

[25] Zeidner M, Matthews G, Shemesh DO. Cognitive-social sources of wellbeing: differentiating the roles of coping style, social support and emotional intelligence. J Happiness Stud [Internet]. 2015 Dec 11;17(6):2481–501. doi: 10.1007/s10902-015-9703-z

[26] Wang J, Mann F, Lloyd-Evans B, Ma R, Johnson S. Associations between loneliness and perceived social support and outcomes of mental health problems: a systematic review. BMC Psychiatry. 2018 May 29;18(1):156. doi: 10.1186/s12888-018-1736-5 ; PMCID: PMC5975705. 29843662 PMC5975705

[27] Alegría M, Álvarez K, DiMarzio K. Immigration and mental health. Curr Epidemiol Rep. 2017 Jun;4(2):145–155. doi: 10.1007/s40471-017-0111-2 Epub 2017 Apr 22. ; PMCID: PMC5966037. 29805955 PMC5966037

[28] Awuah RB, de-Graft Aikins A, Dodoo FN, Meeks KA, Beune EJ, Klipstein-Grobusch K, et al. Psychosocial stressors among Ghanaians in rural and urban Ghana and Ghanaian migrants in Europe. J Health Psychol. 2020 Oct 20:1359105320963549. doi: 10.1177/1359105320963549 Epub ahead of print. . 33081514 PMC8191584

[29] Newman A, Nielsen I, Smyth R, Hirst G. Mediating role of psychological capital in the relationship between social support and wellbeing of refugees. Int Migr [Internet]. 2017 Dec 5;56(2):117–32. doi: 10.1111/imig.12415

[30] Lourel M., Hartmann A, Closon C, Mouda F, Petric-Tatu O. Social support and health: an overview of selected theoretical models for adaptation. In Chen S, editor. Social support, gender and culture, and health benefits Hauppauge: Nova Science Publishers; 2013. pp. 1–20.

[31] Moher D, Liberati A, Tetzlaff J, Altman DG; PRISMA Group. Preferred reporting items for systematic reviews and meta-analyses: the PRISMA statement. PLoS Med. 2009 Jul 21;6(7):e1000097. doi: 10.1371/journal.pmed.1000097 Epub 2009 Jul 21. ; PMCID: PMC2707599. 19621072 PMC2707599

[32] Wells G., Shea B, O’Connell D, Peterson J, Welch V, Losos M, et al. [Internet]. The Newcastle–Ottawa Scale (NOS) for assessing the quality of non-randomized studies in meta-analysis. 2021 [cited 2021 May 8]. Available from http://www.ohri.ca/programs/clinical_epidemiology/oxford.asp

[33] Modesti PA, Reboldi G, Cappuccio FP, Agyemang C, Remuzzi G, Rapi S, et al. Panethnic differences in blood pressure in Europe: A systematic review and meta-analysis. PLoS One. 2016 Jan 25;11(1):e0147601. doi: 10.1371/journal.pone.0147601 ; PMCID: PMC4725677. 26808317 PMC4725677

[34] Joanna Briggs Institute. [Internet]. Checklist for systematic reviews and research synthesis. 2020 [cited 2021 May 8]. Available from https://jbi.global/critical-appraisal-tools

[35] DerSimonian R, Laird N. Meta-analysis in clinical trials. Control Clin Trials. 1986 Sep;7(3):177–88. doi: 10.1016/0197-2456(86)90046-2 . 3802833

[36] Mantel N, Haenszel W. Statistical aspects of the analysis of data from retrospective studies of disease. J Natl Cancer Inst. 1959 Apr;22(4):719–48. . 13655060

[37] Rothman KJ, Boice JD. Epidemiologic analysis with a programmable calculator. Washington DC: US Government Printing Office; 1979.

[38] Daly A, Carey RN, Darcey E, Chih H, LaMontagne AD, Milner A, et al. Workplace psychosocial stressors experienced by migrant workers in Australia: A cross-sectional study. PLoS One. 2018 Sep 20;13(9):e0203998. doi: 10.1371/journal.pone.0203998 ; PMCID: PMC6147467. 30235255 PMC6147467

[39] Daly A, Carey RN, Darcey E, Chih H, LaMontagne AD, Milner A, et al. Using three cross-sectional surveys to compare workplace psychosocial stressors and associated mental health status in six migrant groups working in Australia compared with Australian-born workers. Int J Environ Res Public Health. 2019 Feb 28;16(5):735. doi: 10.3390/ijerph16050735 ; PMCID: PMC6427607. 30823505 PMC6427607

[40] Chen W, Wu S, Ling L, Renzaho AMN. Impacts of social integration and loneliness on mental health of humanitarian migrants in Australia: evidence from a longitudinal study. Aust N Z J Public Health [Internet]. 2019 Jan 2;43(1):46–55. doi: 10.1111/1753-6405.12856 30602072

[41] Adebayo B, Nichols P, Albrecht MA, Brijnath B, Heslop K. Investigating the Impacts of Acculturation Stress on Migrant Care Workers in Australian Residential Aged Care Facilities. J Transcult Nurs. 2020 Aug 10:1043659620947810. doi: 10.1177/1043659620947810 Epub ahead of print. . 32772896

[42] Liu X, Bowe SJ, Li L, Too LS, LaMontagne AD. Psychosocial job characteristics and mental health: Do associations differ by migrant status in an Australian working population sample? PLoS One. 2020 Nov 30;15(11):e0242906. doi: 10.1371/journal.pone.0242906 ; PMCID: PMC7703972. 33253270 PMC7703972

[43] Anjara SG, Nellums LB, Bonetto C, Van Bortel T. Stress, health and quality of life of female migrant domestic workers in Singapore: a cross-sectional study. BMC Womens Health. 2017 Oct 10;17(1):98. doi: 10.1186/s12905-017-0442-7 ; PMCID: PMC5634837. 29017558 PMC5634837

[44] Van Bortel T, Martin S, Anjara S, Nellums LB. Perceived stressors and coping mechanisms of female migrant domestic workers in Singapore. PLoS One. 2019 Mar 20;14(3):e0210717. doi: 10.1371/journal.pone.0210717 ; PMCID: PMC6426224. 30893317 PMC6426224

[45] Baig RB, Chang C-W. Formal and informal social support systems for migrant domestic workers. American Behavioral Scientist [Internet]. 2020 Mar 14;64(6):784–801. doi: 10.1177/0002764220910251

[46] C Y Yeung N, Huang B, Lau CYK, Lau JTF. Feeling Anxious amid the COVID-19 Pandemic: Psychosocial Correlates of Anxiety Symptoms among Filipina Domestic Helpers in Hong Kong. Int J Environ Res Public Health. 2020 Nov 3;17(21):8102. doi: 10.3390/ijerph17218102 ; PMCID: PMC7662612. 33153082 PMC7662612

[47] Capasso R, Zurlo MC, Smith AP. Stress in factory workers in Italy: An application of the ethnicity and work-related stress model in Moroccan factory workers. Psychology and Developing Societies [Internet]. 2018 Jul 24;30(2):199–233. doi: 10.1177/0971333618783397

[48] Gambaro E, Mastrangelo M, Sarchiapone M, Marangon D, Gramaglia C, Vecchi C, et al. Resilience, trauma, and hopelessness: protective or triggering factor for the development of psychopathology among migrants? BMC Psychiatry [Internet]. 2020 Jul 8;20(1). doi: 10.1186/s12888-020-02729-3 32641011 PMC7346618

[49] Crocker R. Emotional testimonies: an ethnographic study of emotional suffering related to migration from Mexico to Arizona. Front Public Health. 2015 Jul 13;3:177. doi: 10.3389/fpubh.2015.00177 ; PMCID: PMC4500103. 26217657 PMC4500103

[50] Organista KC, Jung W, Neilands TB. Working and living conditions and psychological distress in Latino migrant day laborers. Health Educ Behav. 2019 Aug;46(4):637–647. doi: 10.1177/1090198119831753 Epub 2019 Mar 2. . 30829088

[51] Ronda-Pérez E, Martínez JM, Reid A, Agudelo-Suárez AA. Longer residence of Ecuadorian and Colombian migrant workers in Spain associated with new episodes of common mental disorders. Int J Environ Res Public Health. 2019 Jun 6;16(11):2027. doi: 10.3390/ijerph16112027 ; PMCID: PMC6604003. 31174399 PMC6604003

[52] González-Castro JL, Ubillos Landa S, Puente Martínez A, Vera Perea M. The role of emotional intelligence and sociocultural adjustment on migrants’ self-reported mental well-being in Spain: A 14 month follow-up study. Int J Environ Res Public Health. 2020 Feb 13;17(4):1206. doi: 10.3390/ijerph17041206 ; PMCID: PMC7068327. 32069983 PMC7068327

[53] Hatch SL, Gazard B, Williams DR, Frissa S, Goodwin L; SELCoH Study Team, et al. Discrimination and common mental disorder among migrant and ethnic groups: findings from a South East London Community sample. Soc Psychiatry Psychiatr Epidemiol. 2016 May;51(5):689–701. doi: 10.1007/s00127-016-1191-x Epub 2016 Feb 13. ; PMCID: PMC4846681. 26875153 PMC4846681

[54] Martynowska K, Korulczyk T, Mamcarz PJ. Perceived stress and well-being of Polish migrants in the UK after Brexit vote. PLoS One. 2020 Jul 23;15(7):e0236168. doi: 10.1371/journal.pone.0236168 ; PMCID: PMC7377441. 32702031 PMC7377441

[55] Attal JH, Lurie I, Neumark Y. A rapid assessment of migrant careworkers’ psychosocial status during Israel’s COVID-19 lockdown. Isr J Health Policy Res. 2020 Nov 2;9(1):61. doi: 10.1186/s13584-020-00422-0 ; PMCID: PMC7605873. 33138855 PMC7605873

[56] Dhungana RR, Aryal N, Adhikary P, Kc RK, Regmi PR, Devkota B, et al. Psychological morbidity in Nepali cross-border migrants in India: a community based cross-sectional study. BMC Public Health. 2019 Nov 15;19(1):1534. doi: 10.1186/s12889-019-7881-z ; PMCID: PMC6858657. 31730454 PMC6858657

[57] Hong J, Lee J. Decomposing income-related inequalities in self-reported depression and self-rated health among married immigrants in South Korea. Int J Environ Res Public Health. 2019 May 27;16(10):1869. doi: 10.3390/ijerph16101869 ; PMCID: PMC6571644. 31137860 PMC6571644

[58] Htay MNN, Latt SS, Maung KS, Myint WW, Moe S. Mental well-being and its associated factors among Myanmar migrant workers in Penang, Malaysia. Asia Pac J Public Health. 2020 Sep-Oct;32(6–7):320–327. doi: 10.1177/1010539520940199 Epub 2020 Jul 16. . 32672053

[59] Kesornsri S, Sitthimongkol Y, Punpuing S, Vongsirimas N, Hegadoren KM, et al. Mental health and related factors among migrants from Myanmar in Thailand. Journal of Population and Social Studies [Internet]. 2019 Mar 28;27(2):124–38. doi: 10.25133/jpssv27n2.008

[60] Miller R, Ong KIC, Choi S, Shibanuma A, Jimba M. Seeking connection: a mixed methods study of mental well-being and community volunteerism among international migrants in Japan. BMC Public Health [Internet]. 2020 Aug 20;20(1). doi: 10.1016/j.amp.2020.06.001 32819356 PMC7441705

[61] Straiton ML, Aambø AK, Johansen R. Perceived discrimination, health and mental health among immigrants in Norway: the role of moderating factors. BMC Public Health. 2019 Mar 20;19(1):325. doi: 10.1186/s12889-019-6649-9 ; PMCID: PMC6425660. 30894173 PMC6425660

[62] Tilahun M, Workicho A, Angaw DA. Common mental disorders and its associated factors and mental health care services for Ethiopian labour migrants returned from Middle East countries in Addis Ababa, Ethiopia. BMC Health Serv Res. 2020 Jul 23;20(1):681. doi: 10.1186/s12913-020-05502-0 ; PMCID: PMC7376707. 32703193 PMC7376707

[63] Urzúa A, Leiva-Gutiérrez J, Caqueo-Urízar A, Vera-Villarroel P. Rooting mediates the effect of stress by acculturation on the psychological well-being of immigrants living in Chile. PLoS One. 2019 Aug 13;14(8):e0219485. doi: 10.1371/journal.pone.0219485 Erratum in: One PLoS. 2020 Oct 29;15(10):e0241873. ; PMCID: PMC6692025. 31408469 PMC6692025

[64] Chen H, Wang L, Wei Y, Ye B, Dai J, Gao J, et al. The potential psychological mechanism of subjective well-being in migrant workers: a structural equation models analysis. Int J Environ Res Public Health. 2019 Jun 24;16(12):2229. doi: 10.3390/ijerph16122229 ; PMCID: PMC6617093. 31238592 PMC6617093

[65] van de Beek MH, van der Krieke L, Schoevers RA, Veling W. Social exclusion and psychopathology in an online cohort of Moroccan-Dutch migrants: Results of the MEDINA-study. PLoS One. 2017 Jul 10;12(7):e0179827. doi: 10.1371/journal.pone.0179827 ; PMCID: PMC5503196. 28692653 PMC5503196

[66] Lindert J, Ehrenstein OS, Priebe S, Mielck A, Brähler E. Depression and anxiety in labor migrants and refugees—a systematic review and meta-analysis. Soc Sci Med. 2009 Jul;69(2):246–57. doi: 10.1016/j.socscimed.2009.04.032 Epub 2009 Jun 17. . 19539414

[67] Peconga EK, Høgh Thøgersen M. Post-traumatic stress disorder, depression, and anxiety in adult Syrian refugees: What do we know? Scand J Public Health. 2020 Nov;48(7):677–687. doi: 10.1177/1403494819882137 Epub 2019 Dec 8. . 31814514

[68] Close C, Kouvonen A, Bosqui T, Patel K, O’Reilly D, Donnelly M. The mental health and wellbeing of first generation migrants: a systematic-narrative review of reviews. Global Health. 2016 Aug 25;12(1):47. doi: 10.1186/s12992-016-0187-3 ; PMCID: PMC4997738. 27558472 PMC4997738

[69] Doki S, Sasahara S, Matsuzaki I. Stress of working abroad: a systematic review. Int Arch Occup Environ Health. 2018 Oct;91(7):767–784. doi: 10.1007/s00420-018-1333-4 Epub 2018 Jul 2. ; PMCID: PMC6132646. 29967924 PMC6132646

[70] Schutte NS, Malouff JM, Thorsteinsson EB, Bhullar N, Rooke SE. A meta-analytic investigation of the relationship between emotional intelligence and health. Personality and Individual Differences [Internet]. 2007 Apr;42(6):21–33. doi: 10.1016/j.paid.2006.09.003

[71] Martins A, Ramalho N, Morin E. A comprehensive meta-analysis of the relationship between Emotional Intelligence and health. Personality and Individual Differences [Internet]. 2010 Oct;49(6):554–64. doi: 10.1016/j.paid.2010.05.029

[72] Hu T, Zhang D, Wang J. A meta-analysis of the trait resilience and mental health. Personality and Individual Differences [Internet]. 2015 Apr;76:18–27. doi: 10.1016/j.paid.2014.11.039

[73] Oliver J, Brough P. Cognitive appraisal, negative affectivity and psychological well-being. New Zealand Journal of Psychology. 2002 June;31(1):2–7.

[74] de Moor EL, Denollet J, Laceulle OM. Social inhibition, sense of belonging and vulnerability to internalizing problems. J Affect Disord. 2018 Jan 1;225:207–213. doi: 10.1016/j.jad.2017.08.034 Epub 2017 Aug 16. . 28837955

[75] Timmermans I, Versteeg H, Duijndam S, Graafmans C, Polak P, Denollet J. Social inhibition and emotional distress in patients with coronary artery disease: The Type D personality construct. J Health Psychol. 2019 Dec;24(14):1929–1944. doi: 10.1177/1359105317709513 Epub 2017 Jun 18. ; PMCID: PMC6749748. 28810489 PMC6749748

[76] American Psychiatric Association. Diagnostic and statistical manual of mental disorders. 5th ed. Arlington: American Psychiatric Association; 2013.

[77] Borrell-Carrió F, Suchman AL, Epstein RM. The biopsychosocial model 25 years later: principles, practice, and scientific inquiry. Ann Fam Med. 2004 Nov-Dec;2(6):576–82. doi: 10.1370/afm.245 ; PMCID: PMC1466742. 15576544 PMC1466742

[78] Lee W, Neo A, Tan S, Cook AR, Wong ML, Tan J, et al. Health-seeking behaviour of male foreign migrant workers living in a dormitory in Singapore. BMC Health Serv Res. 2014 Jul 10;14:300. doi: 10.1186/1472-6963-14-300 ; PMCID: PMC4097050. 25011488 PMC4097050

[79] Asampong E, Dwuma-Badu K, Stephens J, Srigboh R, Neitzel R, Basu N, et al. Health seeking behaviours among electronic waste workers in Ghana. BMC Public Health [Internet]. 2015 Oct 16;15(1). doi: 10.1186/s12889-015-2376-z 26474859 PMC4609051

[80] H. M. Zehadul Karim A, Mohamad Diah N. Health seeking behavior of the Bangladeshi migrant workers in Malaysia: some suggestive recommendations in adjustive context. Asian Social Science [Internet]. 2015 Apr 20;11(10). doi: 10.5539/ass.v11n10p348

[81] Millán-Franco M, Gómez-Jacinto L, Hombrados-Mendieta I, González-Castro F, García-Cid A. The effect of length of residence and geographical origin on the social inclusion of immigrants. Psychosocial Intervention [Internet]. 2019 Nov;28(3):119–30. doi: 10.5093/pi2019a10

[82] Jurado D, Alarcón RD, Martínez-Ortega JM, Mendieta-Marichal Y, Gutiérrez-Rojas L, Gurpegui M. Factors associated with psychological distress or common mental disorders in migrant populations across the world. Rev Psiquiatr Salud Ment. 2017 Jan-Mar;10(1):45–58. English, Spanish. doi: 10.1016/j.rpsm.2016.04.004 Epub 2016 Jun 10. . 27291831

[83] OECD. Settling In 2018: Indicators of immigrant integration. [Internet]. 2019 [cited 2021 October 26]. Available from doi: 10.1787/9789264307216-8-en

[84] Rudiger A, Spencer S. The economic and social aspect migration. [Internet]. 2003 [cited 2021 October 26]. Available from: https://www.oecd.org/migration/mig/15516956.pdf

[85] Kelloway EK, & Barling J. Job characteristics, role stress and mental health. Journal of Occupational Psychology. 1991;64(4):291–304. doi: 10.1111/j.2044-8325.1991.tb00561.x

[86] Mark G, Smith AP. Effects of occupational stress, job characteristics, coping, and attributional style on the mental health and job satisfaction of university employees. Anxiety Stress Coping. 2012 Jan;25(1):63–78. doi: 10.1080/10615806.2010.548088 Epub 2011 May 24. . 21271408

[87] Mark G, Smith AP. Occupational stress, job characteristics, coping, and the mental health of nurses. Br J Health Psychol. 2012 Sep;17(3):505–21. doi: 10.1111/j.2044-8287.2011.02051.x Epub 2011 Sep 14. . 22107162

[88] Cottini E, Lucifora C. Mental health and working conditions in Europe. ILR Review [Internet]. 2013 Jul;66(4):958–88. doi: 10.1177/001979391306600409

[89] Loher BT, Noe RA, Moeller NL, Fitzgerald MP. A meta-analysis of the relation of job characteristics to job satisfaction. Journal of Applied Psychology. 1985;70(2):280–289. doi: 10.1037/0021-9010.70.2.280

[90] Thomas A, Buboltz WC, Winkelspecht CS. Job characteristics and personality as predictors of job satisfaction. Organizational Analysis. 2004;12(2):205–219. doi: 10.1108/eb028993

[91] Ozturk AB, Hancer M, Im JY. Job Characteristics, Job satisfaction, and organizational commitment for hotel workers in Turkey. Journal of Hospitality Marketing & Management [Internet]. 2014 Feb 25;23(3):294–313. doi: 10.1080/19368623.2013.796866

[92] Connor JB, Miller AM. Occupational stress and adaptation of immigrant nurses from the Philippines. Journal of Research in Nursing [Internet]. 2014 Jun 9;19(6):504–15. doi: 10.1177/1744987114536570

[93] Schilgen B, Handtke O, Nienhaus A, Mösko M. Work-related barriers and resources of migrant and autochthonous homecare nurses in Germany: A qualitative comparative study. Appl Nurs Res. 2019 Apr;46:57–66. doi: 10.1016/j.apnr.2019.02.008 Epub 2019 Feb 12. . 30853077

[94] International Labour Organization. Protecting migrant domestic workers: The international legal framework at a glance. [Internet]. 2016 [cited 2021 October 24]. Available from: https://www.ilo.org/wcmsp5/groups/public/—ed_protect/—protrav/—migrant/documents/briefingnote/wcms_467722.pdf

[95] International Labour Organization. Safe and healthy working environments free from violence and harassment. 2020 [cited 2021 October 24]. Available from: https://www.ilo.org/wcmsp5/groups/public/—ed_protect/—protrav/—safework/documents/publication/wcms_751832.pdf

[96] Liu X, Bowe SJ, Milner A, Li L, Too LS, LaMontagne AD. Job insecurity: A comparative analysis between migrant and native workers in Australia. Int J Environ Res Public Health. 2019 Oct 28;16(21):4159. doi: 10.3390/ijerph16214159 ; PMCID: PMC6861924. 31661926 PMC6861924

[97] Jamil R, Dutta U. Centering the Margins: The precarity of Bangladeshi low-income migrant workers during the time of COVID-19. American Behavioral Scientist [Internet]. 2021 Mar 17; doi: 10.1177/00027642211000397 000276422110003. PMC7969852 38603032

[98] Leigh-Hunt N, Bagguley D, Bash K, Turner V, Turnbull S, Valtorta N, et al. An overview of systematic reviews on the public health consequences of social isolation and loneliness. Public Health. 2017 Nov;152:157–171. doi: 10.1016/j.puhe.2017.07.035 Epub 2017 Sep 12. . 28915435

[99] Ge L, Yap CW, Ong R, Heng BH. Social isolation, loneliness and their relationships with depressive symptoms: A population-based study. PLoS One. 2017 Aug 23;12(8):e0182145. doi: 10.1371/journal.pone.0182145 ; PMCID: PMC5568112. 28832594 PMC5568112

[100] Saeri AK, Cruwys T, Barlow FK, Stronge S, Sibley CG. Social connectedness improves public mental health: Investigating bidirectional relationships in the New Zealand attitudes and values survey. Aust N Z J Psychiatry. 2018 Apr;52(4):365–374. doi: 10.1177/0004867417723990 Epub 2017 Aug 12. . 28803484

[101] Sun J, Sun R, Jiang Y, Chen X, Li Z, Ma Z, et al. The relationship between psychological health and social support: Evidence from physicians in China. PLoS One. 2020 Jan 29;15(1):e0228152. doi: 10.1371/journal.pone.0228152 ; PMCID: PMC6988930. 31995601 PMC6988930

[102] Labrague LJ, De Los Santos JAA. COVID-19 anxiety among front-line nurses: Predictive role of organisational support, personal resilience and social support. J Nurs Manag. 2020 Oct;28(7):1653–1661. doi: 10.1111/jonm.13121 Epub 2020 Aug 21. ; PMCID: PMC7436313. 32770780 PMC7436313

[103] Hennein R, Mew EJ, Lowe SR. Socio-ecological predictors of mental health outcomes among healthcare workers during the COVID-19 pandemic in the United States. PLoS One. 2021 Feb 5;16(2):e0246602. doi: 10.1371/journal.pone.0246602 ; PMCID: PMC7864435. 33544761 PMC7864435

[104] World Health Organization. Mental health promotion and mental health care in refugees and migrants. Copenhagen: WHO Regional Office for Europe; 2018.

[105] Harnois G, Gabriel P. Mental health and work: impact, issues and good practices. Geneva: World Health Organization; 2000.

