# Supplementary material for: Prevalence of common mental health issues among migrant workers: A systematic review and meta-analysis
Source: PLoS One. 2021 Dec 2;16(12):e0260221. doi: 10.1371/journal.pone.0260221 (PMC8638981; doi:10.1371/journal.pone.0260221)
Supplement: S1 Table — (DOCX) [file pone.0260221.s002.docx]

**S1 Table. Study characteristics.**

| No | Authors | Study  Design | Country | | Participant | | Measurement  Tool | Outcome Measurement of the Study | Major findings |
| --- | --- | --- | --- | --- | --- | --- | --- | --- | --- |
|  |  |  |  |  | Number | Age |  |  |  |
| 1 | Adebayo et al. 2020) | Cross sectional | Australia | | 272 | 18 years and older | Riverside Acculturation Stress Inventory (RASI), Depression Anxiety Stress Scale (DASS 21) | Acculturation stress, depression, anxiety, and stress | Mean RASI = 38.4 (SD = 14.1)  85% - 93% in the normal to mild ranges of DASS-21 subscales |
| 2 | Anjara et al. (2017) | Cross sectional | Singapore | | 182 | 18 years and older | The Friendship Scale, Theory X and Theory Y Questionnaire, WHOQoL-Bréf | Social connectivity and overall satisfaction with health and quality of life | Majority (73.6%) of participants showed an overall good or very good quality of life  Age and working experience were found to be significantly associated with overall quality of life, psychological, social and environmental health |
| 3 | Attal et al. (2020) | Cross-sectional | Israel | | 307 | 25 to 65 years old | Hopkins Symptom Checklist-10 (HSCL-10) | Depression and anxiety | 39.1% scored above the overall HSCL-10 threshold, 28.0% were symptomatic on the anxiety subscale, and 38.1% were symptomatic on the depression subscale |
| 4 | Baig & Chang (2020) | Mixed method | Hong Kong | | 2017 | 20 to 40 years old | A series of questions and interviews | Help-seeking preferences and behaviours | Migrant domestic workers preferred to seek for formal support when the issue is employment related and informal support to cope with emotional needs |
| 5 | Yeung et al. (2020) | Cross sectional | Hong Kong | | 295 | 18 years and older | General Anxiety Disorder-7 (GAD-7), Effort–Reward Imbalance Scale, Cancer Perceived Agents of Social Support (CPASS) | Anxiety symptoms, workload during COVID-19 and social support | Participants who reported higher levels of insufficiency of preventive equipment (OR = 1.54, 95%CI: 1.22, 1.95), workload during COVID-19 (OR = 1.95, 95%CI: 1.41, 2.71), worries about being fired if getting COVID-19 (OR = 1.43, 95%CI: 1.18, 1.73), and worries about getting COVID-19 (OR = 1.43, 95%CI: 1.09, 1.87) were associated with a greater risk for probable anxiety. |
| 6 | Capasso et al. (2018) | Cross-sectional | Italy | | 250 | M = 40.78 | Coping Style Inventory, Job Content Questionnaire, Job Satisfaction Scale, Symptom Checklist 90 | Individual characteristics and cultural dimensions, work characteristics and subjective reports of health | Emotional–relational coping, a search identity/  adoption of the host culture and perceived racial discrimination were associated  with lower perception of job satisfaction |
| 7 | Chen et al. (2019) | Cross-sectional | China | | 2573 | 18 to 58 years old | Patient Health Questionnaire-9 (PHQ-9), Personal Wellbeing Index (PWI), Sense of Coherence (SOC) Scale | Depression, subjective well-being (SWB), sense of coherence of migrants (SOC) | SOC showed a positive correlation with SWB (r = 0.46, p < 0.001) and a negative correlation to PHQ (r = −0.53, p < 0.001) |
| 8 | Chen et al. (2019) | Longitudinal | Australia | | 1723 | 18 years and older | PTSD-8 scale, Australian K6 | General health, post-traumatic stress disorder (PTSD), severe mental illness (SMI) | Humanitarian migrants (HM) with increased social integration stressors reported poor general health (aOR:1.56, 95%CI:1.19–2.03); PTSD (aOR:1.67; 95%CI: 1.32–2.13); and SMI (aOR: 1.46; 95%CI: 1.15–1.86)  HMs who reported  overcoming loneliness (aOR:1.50, 95%CI: 1.24–1.83 for SMI and aOR:1.51; 95%CI: 1.22–1.86 for  PTSD) and persistent loneliness (aOR:1.99; 95%CI: 1.51–2.61 for SMI) reported poorer mental  health over time |
| 9 | Crocker (2015) | Qualitative (Ethnographic) | | United States of America | 40 | M = 42 | Digital audio recorder, notes regarding non-verbal cues to emotion | Emotional health, factors intersecting with the emotional health of individual migrants before, during, and after the process of migration | Emotional hardship among immigrants is trauma (50%), fear (65%), depression (75%), loneliness (75%), sadness (80%), and stress (85%) |
| 10 | Daly et al. (2018) | Cross-sectional | Australia | | 585 | 18 to 65 years old | General Ethnic Discrimination Scale (GEDS), 10 items assessing job complexity, job control  and job security | Ethnic discrimination, psychosocial job quality | 82.9% participants reported experiencing at least one of the workplace psychosocial stressors  Overall psychosocial job quality decreased with education and was associated with  occupation type which interacted with ethnicity and gender |
| 11 | Daly et al. (2019) | Cross-sectional | Australia | | 2215 | 18 to 65 years old | Mental Health Inventory (MHI5), Kessler 6 (K6) | Mental health problem, psychological distress | Overall job  adversity, working in complex or demanding jobs, and jobs with low security were associated with  probable mental health problems in all workers  Workers from Arabic-speaking countries had the highest risk of mental health problems when they felt they were unfairly paid (OR = 2.0) |
| 12 | Dhungana et al. (2019) | Cross-sectional | India | | 751 | M= 31.5 | General Health Questionnaire (GHQ)-12 | Psychological morbidity | The prevalence of psychological morbidity was 13.5% (CI: 11.2–16.1%) |
| 13 | Gambaro et al. (2020) | Cross-sectional | Italy | | 119 | 18 to 79 years old | Zung Self-Rating Depression Scale (SDS), Zung Self-Rating Anxiety Scale (SAS), Childhood Trauma Questionnaire (CTQ), Posttraumatic Stress Disorder Checklist for DSM-5 (PCL-5), Brief Aggression Questionnaire (BAQ) | Depression, anxiety, childhood or adolescent abuse and neglect, post-traumatic stress disorder (PTSD) symptoms | 53.39% of migrants scored above the PCL-5 cut-off score  SDS scores below the cut-off suggested the presence of depression in 42.37% |
| 14 | González-Castro et al. (2020) | Longitudinal | Spain | | 63 | M = 34 | 12-item General Health Questionnaire (GHQ-12), Trait Meta-Mood Scale (TMMS), 29 item Socio-Cultural Adaptation Scale (SCAS) | Perceived psychological distress, emotional intelligence, socio-cultural adjustment | EI and sociocultural adjustment have an impact on perceived psychological distress (mood repair is a predictor of positive mental well-being; focusing on one’s mood and emotions is an  predictor of negative mental well-being) |
| 15 | Hatch et al. (2016) | Longitudinal | United Kingdom | | 1052 | 16 years old and above | Revised Clinical Interview Schedule (CIS-R) | Common mental disorder (CMD) | The prevalence of CMD symptoms was 22.1 % for the total sample for SELCoH 2, with 12.3 % having a CIS-R score above the cut-off |
| 16 | Hong & Lee (2019) | Cross-sectional | South Korea | | 15,321 | M = 37.8 | Questions used by World Health Organisation | Self-reported depression and self-rated health (SRH) | 34.6% reported experiences of depressive symptoms in the past year, and 9.5% reported their current health to be poor or very poor (weighted) |
| 17 | Htay et al. (2020) | Cross-sectional | Malaysia | | 192 | 18 years old and above | WHO-5 Well-Being Index Scale (WHO-5), Mental Health subscale from the 36-Item Short-Form Health Survey (SF-36) | Wellbeing, mental health | WHO-5 = 79.2% had poor mental well-being |
| 18 | Kesornsri et al. (2019) | Cross-sectional | Thailand | | 445 | 18 to 60 years old | Interpersonal Support Evaluation List (ISEL-12), Acculturative Stress Scale (ASS), Perceived Stress Scale (PSS), Hopkins Symptom Check List (HSCL-25) | Perceived support level, acculturative stress level, stress level, anxiety and depression | Depressive symptom scores were positively related to PSS scores (r = 0.49, p < 0.01) and to ASS scores (r = 0.49, p < 0.01) |
| 19 | Liu et al. (2020) | Cross-sectional | Australia | | 8969 | 15 years old and above | Job-Demand Control Model, Mental Health Inventory-5 score (MHI-5) | Psychosocial job characteristics, mental health | Skill discretion and decision authority  were positively associated with the MHI-5 score while job insecurity was negatively associated with the MHI-5 score |
| 20 | Martynowska et al. (2020) | Cross-sectional | United Kingdom | | 551 | 17 to 64 years old | Stress Scale-Mind Garden, Scale of Psychological Wellbeing, Satisfaction with Life Scale | Perceived stress, psychological wellbeing, life satisfaction | Most of the respondents did not notice any change in the attitude or behaviour of the supervisor (81%) or co-workers (84%). |
| 21 | Miller et al. (2020) | Mixed method | Japan | | 292 | 20 years old and above | Warwick-Edinburgh Mental Well-being Scale (WEMWBS), qualitative interview | Mental well-being, perceptions  of satisfaction with life in the city | Volunteering was not associated with higher mental  well-being score (p = 0.215), but instead, not feeling isolated (p = 0.008), feeling connected to Japan (p = 0.001) and  employment satisfaction (p < 0.001) was significantly associated with mental well-being |
| 22 | Organista et al. (2020) | Cross-sectional | United States | | 344 | 18 years old and above | Center for Epidemiological Studies– Depression Scale (CES-D), Generalized Anxiety Disorder screening instrument (GAD-7) | Depression and anxiety | Direct paths linking working conditions to desesperación (β = −0.10, SE = 0.03, p ≤ .01) and depression (β = −0.11, SE = 0.02, p ≤ .01) are negative  Direct paths linking living conditions to desesperación  (β = −0.19, SE = 0.03, p ≤ .001) and depression  (β = −0.17, SE = 0.02, p ≤ .001) are negative |
| 23 | Ronda-Pérez et al. (2019) | Longitudinal | Spain | | 130 | 18 years old and above | 12-item General Health Questionnaire (GHQ-12) | Common mental disorders | Incidence of common mental disorders is higher in workers born in Spain than immigrants (60.7% compared to 25.0%, respectively; p < 0.001). |
| 24 | Straiton et al. (2019) | Cross-sectional | Norway | | 4294 | 16 to 66 years old | 5-item Hopkins Symptoms Checklist (HSCL) | Mental health problem, perceived discrimination (PD) | 12.7% (n = 545) scored above the cut-off for mental health problems and 71.4% (n = 3060) for good general health |
| 25 | Tilahun et al. (2020) | Mixed method | Ethiopia | | 517 | 18 years old and above | WHO Self Report Questionnaire-20 (SRQ 20) | Mental distress | The prevalence of common mental disorder was found to be 29.2% |
| 26 | Urzúa et al. (2019) | Cross-sectional | Chile | | 684 | 18 to 71 years old | Riff’s Psychological Well-being Scale, stress by acculturation, rooting within the host country | Psychological well-being, stress level, indicator of settling down | The structural model obtained a CFI = .922 and an RMSEA = .042 I.C 90% [. 039 - .044]  The role of rooting in the host location mediated the effect of acculturation stress on mental well-being |
| 27 | Van Bortel et al. (2019) | Qualitative (Thematic analysis) | Singapore | | 182 | 20 to 63 years old | Open-ended questions in the qualitative component | Causes of stress, coping strategies, social support | Participants consistently reported work was a primary source of stress  Participants consistently reported a need for time to themselves in order to cope with the stressors  Participants described that the comfort of company was beneficial |
